# Bone envelope for implant placement after alveolar ridge preservation: a systematic review and meta-analysis

**DOI:** 10.1186/s40729-022-00453-z

**Published:** 2022-12-08

**Authors:** Kai R. Fischer, Alex Solderer, Kristina Arlt, Christian Heumann, Chun Ching Liu, Patrick R. Schmidlin

**Affiliations:** 1 Clinic of Conservative & Preventive Dentistry, Division of Periodontology & Peri-Implant Diseases, Center of Dental Medicine, University of Zurich, Plattenstrasse 11, 8032 Zurich, Switzerland; 2 Department of Statistics, Ludwig-Maximilian-University, Munich, Germany

**Keywords:** Alveolar ridge preservation, Socket healing, Deproteinized bovine bone mineral (DBBM), Dental implant, Guided bone regeneration (GBR), Extraction socket

## Abstract

**Purpose:**

To assess the dimensional establishment of a bony envelope after alveolar ridge preservation (ARP) with deproteinized bovine bone mineral (DBBM) in order to estimate the surgical feasibility of standard diameter implants placement without any additional augmentation methods.

**Methods:**

PubMed, Embase and CENTRAL databases were searched for suitable titles and abstracts using PICO elements. Inclusion criteria were as follows: randomized controlled trials (RCTs) comprising at least ten systemically healthy patients; test groups comprised placement of (collagenated) DBBM w/o membrane and control groups of no grafting, respectively. Selected abstracts were checked regarding their suitability, followed by full-text screening and subsequent statistical data analysis. Probabilities and number needed to treat (NNT) for implant placement without any further need of bone graft were calculated.

**Results:**

The initial database search identified 2583 studies. Finally, nine studies with a total of 177 implants placed after ARP with DBBM and 130 implants after SH were included for the quantitative and qualitative evaluation. A mean difference of 1.13 mm in ridge width in favour of ARP with DBBM could be calculated throughout all included studies (95% CI 0.28–1.98, *t*2 = 1–1063, *I*2 = 68.0%, *p* < 0.01). Probabilities for implant placement with 2 mm surrounding bone requiring theoretically no further bone augmentation ranged from 6 to 19% depending on implant diameter (3.25: 19%, RD = 0.19, *C* = 0.06–0.32, *p* < 0.01/4.0: 14%, RD = 0.14, *C* = 0.05–0.23, *p* < 0.01/5.0: 6%, RD = 0.06, *C* = 0.00–0.12, *p* = 0.06).

**Conclusion:**

ARP employing DBBM reduces ridge shrinkage on average by 1.13 mm and improves the possibility to place standard diameter implants with up to 2 mm circumferential bone housing; however, no ARP would have been necessary or additional augmentative bone interventions are still required in 4 out of 5 cases.

**Graphical Abstract:**

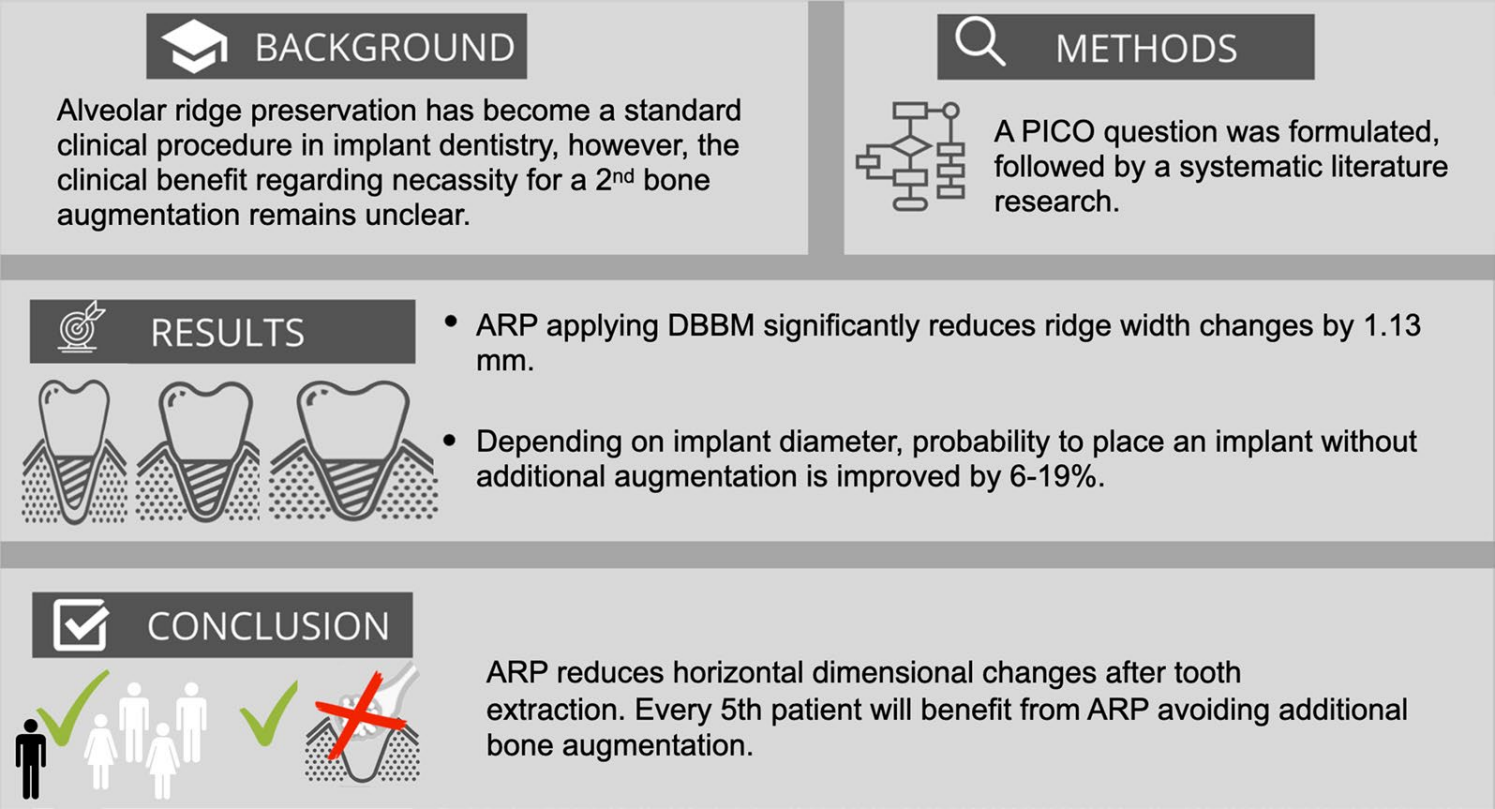

## Background

Dental implant therapy has become a routine procedure when replacing missing teeth, especially if a sufficient bone volume is present. In contrast, a lack of adequate bone width and height represent a lack of bony housing at the time of implant placement, hamper adequate implant placement and render simultaneous or staged bone regeneration measures necessary. These additional measures are costly, clinically demanding and time-consuming, bearing the risk of complications in the short- and long-term [1]. As a preventive consequence, avoiding bone loss at the time of extraction is important to reduce these above-mentioned problems and thus, clinicians are highly sensitized regarding marked alterations of bone volume after tooth extraction. Schropp et al. dramatically illustrated a horizontal bone loss accounting for 5–7 mm within the first 12 months [2], which corresponds to approximately 50% of the original width of the alveolar bone [2]. In an experimental study, the buccal bone wall of the extraction socket came in the focus of these marked remodelling alterations especially is the coronal part of, which has been explained by the presence of functionally inactive bundle bone [3]. Since the so-called bundle bone loses its function as part of the periodontal attachment apparatus after tooth extraction, it will be inevitably resorbed due to osteoclastic activity. This results in a substantial vertical and horizontal reduction of mainly the buccal wall of extraction sites [3]. Especially in the anterior zone, any marked alterations of the extraction socket can jeopardize the aesthetic outcome. Therefore, an effective prevention of a ridge collapse should be prevented or minimized after tooth extraction, leading to more predictable outcomes with improved aesthetics, preferably with fewer surgical procedures. In this context, various methods and materials have been introduced and evaluated to obtain a so-called suitable bony envelope, which ideally limits or even avoids any additional bone augmentation needs after tooth extraction and alveolar bone preservation measures [4, 5].

Several studies have proposed adjusted guided bone preservation techniques following tooth extraction using the placement of graft materials with or without the use of occlusive membranes [6–9]. The classical alveolar ridge preservation technique (ARP) aims to adequately control bone loss over the re-establishing bone contour around and actual bone-neogenesis within the socket mainly avoiding further bone augmentation procedures while trying to achieve equally high implant success rates as implants placed in pristine bone. Implants placed after ARP show similar aesthetic results, but higher implant survival rates compared to immediate implant placement [10]. So far, no technique or biomaterial has proven to be able to entirely maintain the original ridge dimensions yet and the influence on long-term implant success still remains unclear in many aspects [4, 11]. In addition, the principally relevant question not only to the dentist, but also to the patient still remains largely unanswered according to the author’s knowledge, namely: “Is it be possible to place an implant with “sufficient” surrounding bone around implants of a given diameter after ARP without additional bone augmentation measures?” Most clinical studies and reviews so far have measured vertical or horizontal bone dimensions only. However, the need for additional augmentative procedures from a clinical point of view has been mostly neglected. As a clinically demanding requirement, augmentation procedures at the time of extraction make only sense, if they also avoid or significantly reduce the need for additional augmentation at the time of implant placement (i.e. avoiding a staged protocol or any augmentation).

Therefore, the aim of this systematic review was to assess dimensional establishment of a bony envelope after alveolar ridge preservation (ARP) with (collagenated) deproteinized bovine bone mineral (DBBM) without/with membrane in order to estimate the surgical feasibility of standard diameter implants placement without any additional augmentation methods. We hypothesize that ARP improves the possibility—expressed in percentage—to place an implant compared to spontaneous healing (SH).

## Methods

Preferred Reporting Items for Systematic Review and Meta-Analyses (PRISMA) guidelines were followed for this review [12]. The checklist can be found in the Appendix.

### Focused question

The focused questions were:

1. Is there a higher probability for implant placement without additional guided bone regeneration (GBR) for sites with ARP with DBBM compared to sites undergoing SH within a predefined bony housing of 2 mm?
2. Is there a higher probability for the possibility to place an implant simultaneous with only minimal need for GBR for sites with ARP with DBBM compared to sites undergoing SH within a predefined bony housing of 1 mm?

### PICO question

PICO elements were used for online research to ensure adequate and orderly data and information collection:

(P) Population: Patients having tooth extraction.

(I) Intervention: ARP with (collagenated) xenogenic bone substitute material (deproteinized bovine bone mineral, DBBM) in combination without/with a membrane (resorbable/non-resorbable).

(C) Control: Control group with SH.

(O) Outcome: Probability of implant placement without additional GBR or bone augmentation needed.

(S) Study Design: Randomized controlled trials only will be included.

### Study selection criteria

Inclusion criteria:

- Randomized controlled clinical studies (with at least ten participants overall).
- Test group (DBBM ± membrane).
- Control group (without ARP).
- Patients without relevant systemic diseases.
- Publications in English.

Exclusion criteria:

- Animal studies.
- Human studies involving less than ten patients.
- Other graft materials than DBBM.
- No control group with SH available.
- Other language than English.

### Search strategy

Three online databases (PubMed, Embase, CENTRAL) were screened for suitable titles and abstracts from the period from 2013 to August 2022 the online research was carried out by a professional and experienced librarian from the University of Zurich.

At the beginning, search terms were defined, which should be used to screen the online databases for suitable titles and abstracts.

Search terms were as follows:

"socket healing" OR (“socket” OR “ridge” OR “alveolar” OR “bone”) AND (“preserve” OR “augment” OR "guided bone regeneration").

AND

(“bone” OR “xenogenic”) AND (“graft” OR “xenograft” OR “substitute”) OR “DBBM” OR "collagen membrane": OR "deproteinized bovine bone mineral”.

In addition, a hand search of the grey literature was carried out.

### Article selection

Two authors (K.A. and K.F.) independently screened and evaluated the publications by titles and abstracts. Then, available titles and abstracts were collected and discussed before being finally included or excluded. Studies were excluded, if needed raw data were not provided by the authors within four weeks.

### Data extraction

Data were assessed by two authors (K.A. and A.S.) independently. The following key points were collected for the included RCTs and summarized in Table 1: authors, year of publication, number of included patients, compared treatment arms with assessed implant sites, healing and follow-up period.

**Table 1:** Overview of characteristics of the included studies (*n* = 9)

Author and year	Design	No. of patients	Treatment-arms (no. of implants)	Healing period/follow-up
Aimetti et al. 2018 [21]	RCT	30	i.DBBM + collagen and membrane (*n* = 15) ii.Spont. healing (*n* = 15)	12 months
Ben Amara et al. 2021 [23]	RCT	34	i.DBBM + collagen and membrane (*n* = 18) ii.Spont. healing (*n* = 16)	6 months
Iorio-Siciliano et al. 2017 [22]	RCT	20	i.DBBM and membrane (*n* = 10) ii.Spont. healing (*n* = 10)	6 months
Iorio-Siciliano et al. 2020 [16]	RCT	40	i.DBBM + collagen and membrane (*n* = 12) ii.DBBM and membrane (*n* = 13) iii.Spont. healing (*n* = 15)	6 months
Jonker et al. 2021 [18]	RCT	75	i.DBBM + collagen and collagen matrix (n = 25) ii.DBBM + collagen and CTG (n = 25) iii.Spont. healing (*n* = 25)	2 months
Jung et al. 2013 [17]	RCT	40	i.DBBM + collagen and membrane (*n* = 10) ii.Beta-TCP (*n* = 10) iii.DBBM + collagen and CTG (*n* = 10) iv.Spont. healing (n = 10)	6 months
Jung et al. 2018 [15]	RCT split-mouth	18	i.DBBM and membrane (n = 18) ii.Spont. healing (*n* = 18)	3 and 6 months
Machtei et al. 2019 [19]	RCT	33	i.DBBM (*n* = 11) ii.Alloplast (*n* = 11) iii.Spont. healing (*n* = 11)	4 months
Stumbras et al. 2021 [20]	RCT	40	i.DBBM and membrane (*n* = 10) ii.Allograft and membrane (*n* = 10) iii.PRGF (*n* = 10) iv.Spont. healing (*n* = 10)	3 months

DBBM, deproteinized bovine bone mineral; CTG, connective tissue graft; Beta-TCP, beta tricalcium phosphate, PRGF, platelet rich growth factors

For meta-analysis, studies with different treatment group arms including (collagenated) DBBM with or without the additional use of membrane, DBBM groups were taken together and compared as one test group to the control group without ARP measures.

#### Outcome measures

Outcome at the time-point of implant placement was collected. The primary outcome was bone crest width expressed in millimetres (Table 2).

**Table 2:** Excluded studies sorted according to the reason of exclusion at full-text screening (*n* = 77)

Graft choice	Alkanan et al. 2019 Al Qabbani et al. 2018 Barone et al. 2016 Barone et al. 2017 Cavdar et al. 2017 Festa et al.v2013 Kotsakis et al. 2014
No control group with spontaneous healing	Barone et al. 2013 Calasans-Maia et al. 2014 de Carvalho Formiga et al. 2019 Lai et al. 2020 Lim et al. 2017 Llanos et al. 2019 Mercado et al. 2021 Nart et al. 2017 Sadeghi et al. 2016 Santana et al. 2019 Scheyer et al. 2016 Serrano Mйndez et al. 2017 Tomasi et al. 2018
Soft tissue measurements only	Barone et al. 2013 Fickl et al. 2017 Flьgge et al. 2015 Thalmair et al. 2013
Unsuitable data assessed	Amaral et al. 2020 Andre et al. 2021 Attia et al. 2020 (× 2) Barone et al. 2017 (× 2) Block et al. 2020 Botilde et al. 2020 Fischer et al. 2018 Lim et al. 2019 Lin et al. 2022 Natale et al. 2018 Nevins et al. 2019 Noronha et al. 2017 Ranganathan et al. 2017 Pang et al. 2014 Sbordone et al.2017
Under 10 patients	Andrade et al. 2020 Nevins et al. 2018 Parthasaradhi et al. 2015 Shakibaie et al. 2013 Yang and Ouyang 2015
Study design	Cardaropoli et al. 2018 Dubus et al. 2019 Kim et al. 2020 (× 3) Lee et al. 2021 Pang et al. 2017 Resende et al. 2019 Rode et al. 2020
No authors and no results available	Al Hamed et al. 2019 Nct (× 10) Irct2013080414270N 2014
Animal studies	Al Fotawi et al. 2020 Allan et al. 2021
No raw data provided by the authors	Debel et al. 2021 Flores Fraile et al. 2020 Lee et al. 2020 (× 2) Lim et al. 2019 Morelli et al. 2020 Shim et al. 2018

### Data analysis

#### Calculation of probabilities

**Fig. 1:**
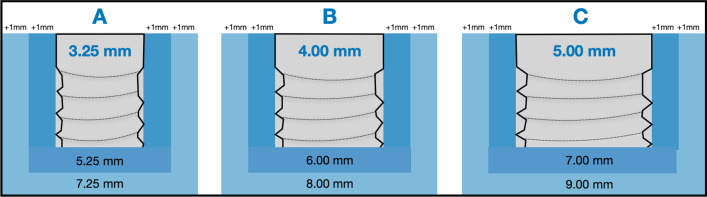
Illustration of the calculations with regard to a theoretical bony housing with circumferential bone thicknesses of either 1 or 2 mm of three different implant diameters (**A**–**C**). Probability calculations were the used accordingly

The required alveolar ridge width in millimetre (mm) was used as a theoretical clinically required value to achieve, i.e. the probability that the observed outcomes have a greater value than the required size was calculated. Standard implant diameters were set at 3.25, 4.0 and 5.0 mm, respectively (Fig. 1).

Assuming normality of the observed outcome (bone crest in mm) and since also the standard deviations SD are estimated by the observed data, a (predictive) t-distribution, centred at the observed bone crest means in the groups, was used (with R Statistical Software, function pt) for calculating the probabilities that the observed outcomes have a size greater than the required one.

Once the probabilities have been calculated, the sample size n of the corresponding group (test or control) was used to calculate an estimated number of events fulfilling the condition (primary outcome *X* > required size), so that the required size is achieved, i.e. the probability was multiplied by the sample size *n*:

Estimated number of events: Events *E* = *n* * *P* (*X* > required). This is done for the experimental (test) group and the control group separately.

The meta-analysis was then based on these event numbers applying a meta-analysis for binary outcomes, where the risk difference (RD) between experimental and control was used as target parameter.

The meta-analytical methods (functions metacont and metabin form R package meta), [13] herein used were as follows:

- A random effects model for the effect size calculation for the primary outcome (bone crest) using the inverse variance method, the Mantel–Haenszel estimator (random effects version) for the dichotomous outcomes based on the above described procedure, both with restricted maximum likelihood estimator for the between study variance tau^2^. As risk measure the risk difference (RD) is used because of its good interpretability;
- I^2^ describes the percentage of the variability in effect estimates that is due to heterogeneity;
- Funnel plots for showing possible reporting bias;
- Numbers needed to treat (NNT) are calculated for the dichotomous analysis using the inverse of the absolute risk difference (1/|RD|).

### Quality assessment

The criteria for the risk of bias assessment followed the Cochrane Collaboration’s tool (2011) and was carried out independently by two reviewers (A.S. & K.F., [14]).

The risk was categorized as low if all criteria were met, moderate if one criterium was missing and high if two or more criteria were missing.

### Risk of bias across studies

The publication bias was evaluated using funnel plots for the outcomes using function funnel from the R package metaphor [13]. A sensitivity analysis of the meta-analysis results was also performed by selectively excluding studies from the different analyses.

## Results

The initial database search was carried out by a librarian from the University of Zurich and yielded 2583 studies. One study was added after hand search of the grey literature. Title and abstract screening leaded to 86 eligible full-texts.

Full-text screening then led to the exclusion of 77 studies as shown in Table 2.

**Fig. 2:**
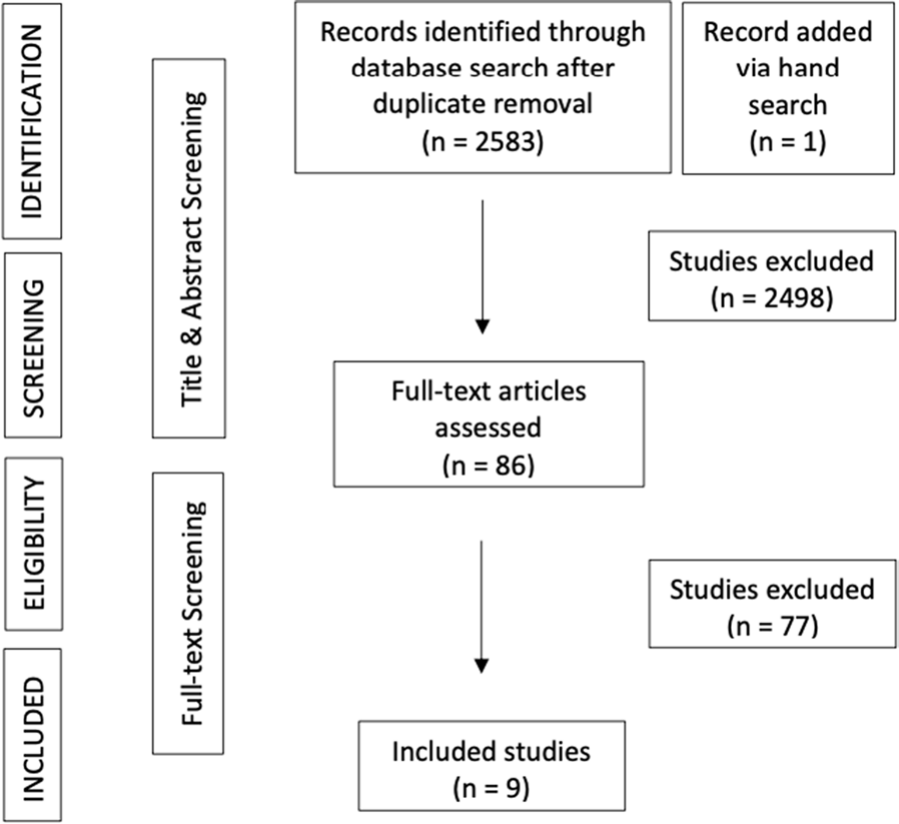
Prisma flowchart

Finally, nine studies were included in the quantitative and qualitative assessment as elucidated in Fig. 2, which shows the PRISMA flowchart.

Inter-examiner agreement of a Cohen's kappa (*K*) of 0.82 was achieved after initial screening. Afterwards full-text screening was done by both authors resulting in a Cohen's kappa (*K*) of 0.76. The authors discussed discrepancies until reaching consent.

### Study characteristics

The general characteristics of the nine included studies are summarized in Table 1.

#### Study design

Eight of the included studies were parallel arm randomized controlled studies, while one study represented a split-mouth design [15]. Of the eight parallel arm studies, five had more than two groups [16–20].

#### Studies population and setting

Eight studies were conducted at a university setting. One study did not declare the study setting [17]. Populations sizes of the included studies ranged from 20 to 75 included patients. While the size of the control and test groups ranged from 10 to 25 patients.

#### Treatment site features

Four studies assessed mixed, lateral and front tooth regions, whereas five studies included anterior teeth sites only. Anterior sites included teeth from canine to canine (3–3). Two studies included maxillary sites only [18, 20], while all others assessed sites in both mandible and maxilla.

#### Biomaterials

All included studies had at least one group with the application of a xenogenic material, using deproteinized bovine bone mineral (DBBM) alone (*n* = 4), in combination with collagen (*n* = 5) and/or a membrane (*n* = 6). DBBM treatment groups have been pooled within the studies from Iorio-Siciliano et al. (2020) [16], Jonker et al. (2021) [17], Jung et al. 2013 [18]. Additionally to the DBBM groups, in one study allografts [20], in two studies alloplasts [17, 19] were used in other intervention groups. One study used platelet rich growth factors (PRGF) [20]. These groups were not assessed in the current review.

#### Follow-up time

The healing period of the ARP before implant placement varied between at least two [18] and maximum twelve [21] months.

#### Measurements

Horizontal bone crest width measurements were taken intra-surgically before implant placement with a calibrated periodontal probe or calliper [16, 22], with prefabricated measuring stents [19] or radiographically using a cone-beam computed tomography (CBCT) scan [15, 17, 18, 20, 21, 23]. Measurements in eight of nine included studies were carried out at 1 mm below or at crest level, while Machtei et al. assessed at 3 mm below the crest margin [19].

### Clinical outcomes

A total of 177 implants were placed after ARP with DBBM and 130 implants after SH were included for the analysis. Measurements are illustrated in Table 3.

**Table 3:** Description of the included treatment-arms with respective outcome measures in terms of achieved bone width

Author	Implant area	Treatment arms	No. of observations	Bone width at implant placement mm (SD)	Healing period/follow-up
Aimetti et al. 2018 [21]	Anterior	DBBM + collagen and membrane Spont. healing	15 15	6.65 (1.41) 3.99 (1.30)	12 months
Ben Amara et al. 2021 [23]	Anterior and molar	DBBM + collagen and membrane Spont. healing	18 16	7.65 (4.45) 4.04 (3.83)	6 months
Iorio-Siciliano et al. 2017 [22]	Anterior and molar	DBBM and membrane Spont. healing	10 10	9.70 (2.30) 9.80 (1.50)	6 months
Iorio-Siciliano et al. 2020 [16]	Anterior and molar	DBBM + collagen and membrane DBBM and membrane Spont. healing	12 13 15	7.80 (1.90) 8.20 (2.10) 8.70 (2.90)	6 months
Jonker et al. 2021 [18]	Anterior	DBBM + collagen and collagen matrix DBBM + collagen and CTG Spont. healing	25 25 25	7.53 (1.37) 7.06 (1.67) 5.68 (2.30)	2 months
Jung et al. 2013 [17]	Anterior	DBBM + collagen and soft-tissue graft DBBM + collagen and membrane Spont. healing	10 10 10	5.79 (2.12) 5.13 (1.50) 5.77 (1.24)	6 months
Jung et al. 2018 [15]	Anterior and molar	DBBM and membrane Spont. healing	18 18	7.73 (3.56) 7.26 (3.89)	3 and 6 months
Machtei et al. 2019 [19]	Anterior	DBBM Spont. healing	11 11	7.25 (1.90) 5.35 (1.20)	4 months
Stumbras et al. 2021 [20]	Anterior	DBBM and membrane Spont. healing	10 10	7.22 (0.86) 5.99 (0.73)	3 months

DBBM = deproteinized bovine bone mineral; CTG = connective tissue graft

#### ARP with DBBM

In anterior sites, crestal width varied from a mean of 5.13 mm (SD = 1.50) to 7.53 mm (SD = 1.37) at implant placement in DBBM groups. Regions including mixed (anterior teeth and molars) sites showed a mean width of 7.65 mm (SD = 4.40) to 9.70 mm (SD = 2.30, Table 3).

#### Spontaneous healing

After SH, the crest width in anterior sites ranged from 3.99 mm (SD = 1.30) to 5.77 mm (SD = 1.24), while mixed sites ranged from 4.04 mm (SD = 3.83, 24) to 9.80 mm (SD = 1.50, 16).

#### Continuous outcome (mean and SD as given in the study)

**Fig. 3:**
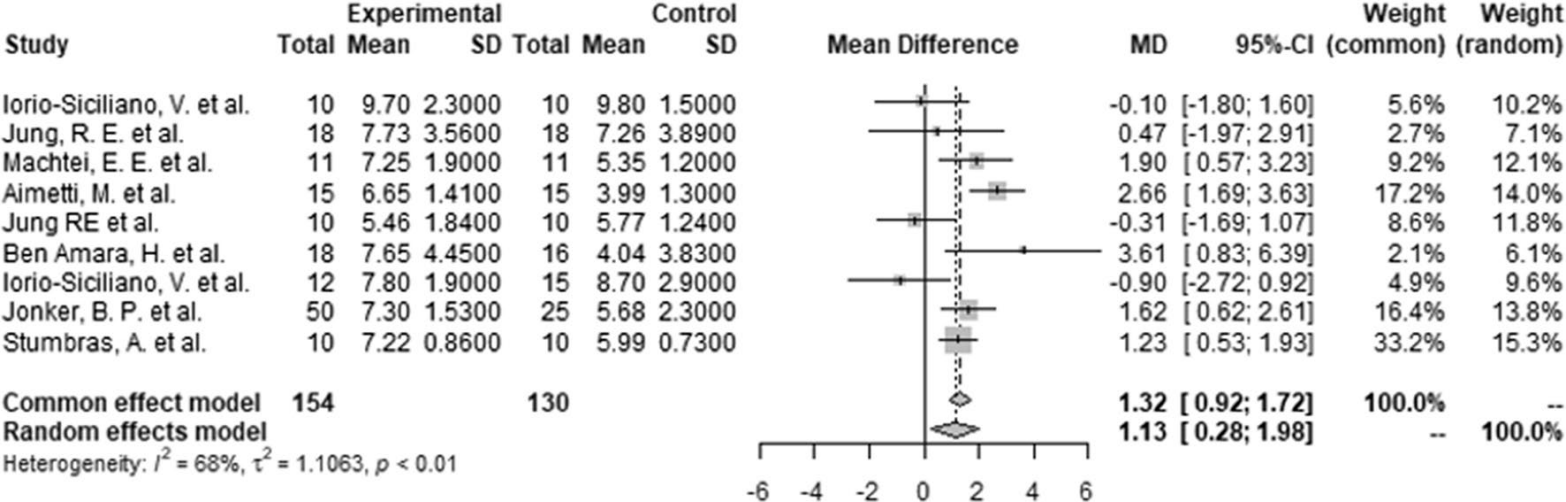
Meta-analysis results for the primary outcome (bone crest in mm) for implant placement in a bone envelope throughout all the studies

There was evidence (MD = 1.13, 95% CI 0.28–1.98, *t*2 = 1–1063, *I*2 = 68.0%) that ARP using DBBM, led to significantly less bone resorption (*p* < 0.01). A mean difference of 1.13 mm in favour of ARP with DBBM could be calculated throughout all included studies (Fig. 3).

#### Probability of standard implant placement

##### Groups: ARP—none, 3.25-mm implants

**Fig. 4:**
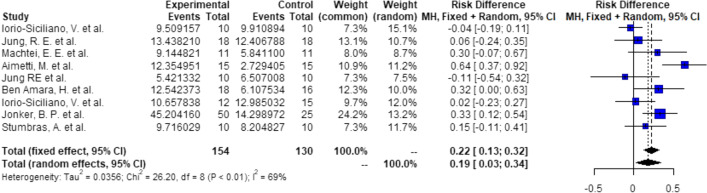
Meta-analysis results for the binary outcomes for implant placement in a bone envelope of 5.25 mm

**Fig. 5:**
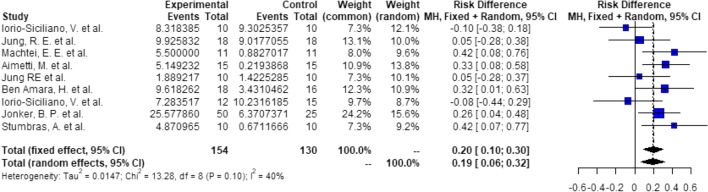
Meta-analysis results for the binary outcomes for implant placement in a bone envelope of 7.25 mm

1. Probabilities/events for 5.25 mm (1 + 3.25 + 1):
  There is a significant higher probability of 19% (RD = 0.19, *C* = 0.03–0.34, *p* = 0.018), that after ARP using DBBM, 3.25 mm implants can be placed with 1 mm of circumferential bony housing allowing simultaneous GBR (Fig. 4).
2. Probabilities/events for 7.25 mm (2 + 3.25 + 2):
  There is a significant higher probability of 19% (RD = 0.19, *C* = 0.06–0.32, *p* < 0.01), that after ARP using DBBM, 3.25 mm implants can be placed without any further bone grafting procedure with 2 mm of circumferential bony housing (Fig. 5).

##### Groups: ARP—none, 4-mm implants

**Fig. 6:**
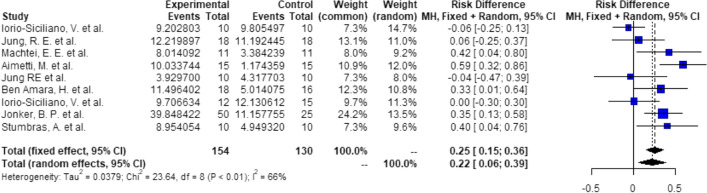
Meta-analysis results for the binary outcomes for implant placement in a bone envelope of 6.0 mm

**Fig. 7:**
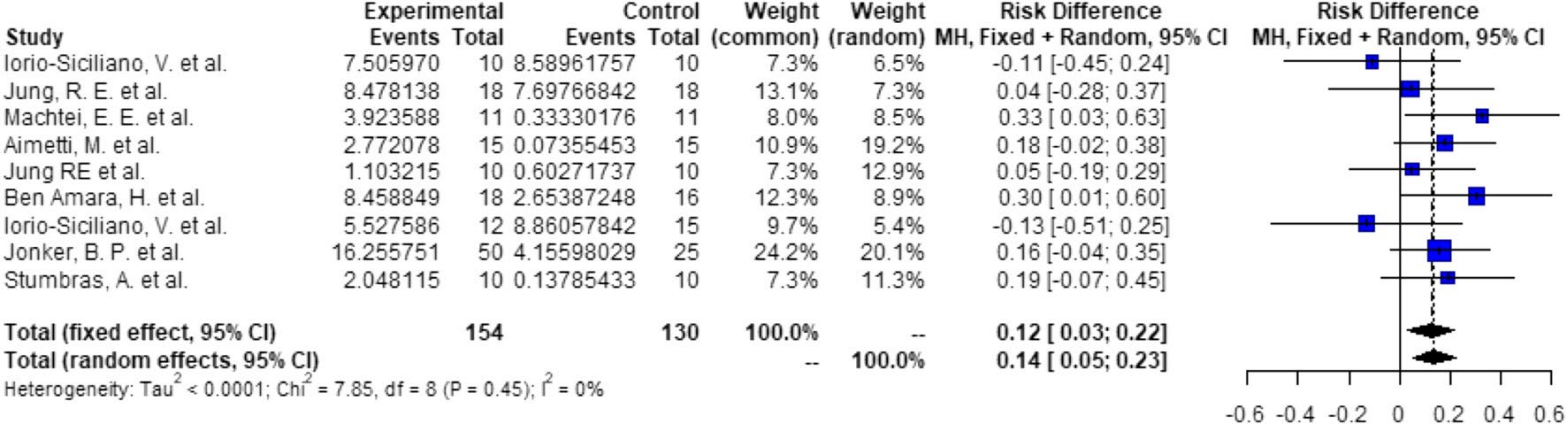
Meta-analysis results for the binary outcomes for implant placement in a bone envelope of 8.0 mm

1. Probabilities/events for 6 (1 + 4 + 1) mm:
  There is a significant higher probability of 22% (RD = 0.22, *C* = 0.06–0.39, *p* < 0.01), that after ARP using DBBM, 4-mm implants can be placed with 1 mm of circumferential bony housing allowing simultaneous GBR (Fig. 6).
2. Probabilities/events for 8 (2 + 4 + 2) mm:
  There is a significant higher probability of 14% (RD = 0.14, *C* = 0.05–0.23, *p* < 0.01), that after ARP using DBBM, 4 mm implants can be placed without any further bone grafting procedure with 2 mm of circumferential bony housing (Fig. 7).

##### Groups: ARP—none, 5-mm implants

**Fig. 8:**
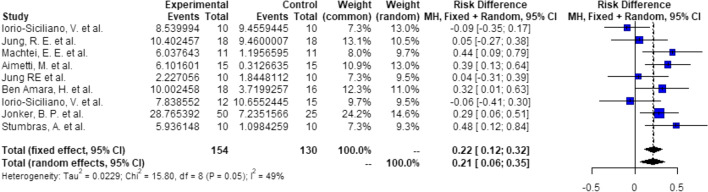
Meta-analysis results for the binary outcomes for implant placement in a bone envelope of 7.0 mm

**Fig. 9:**
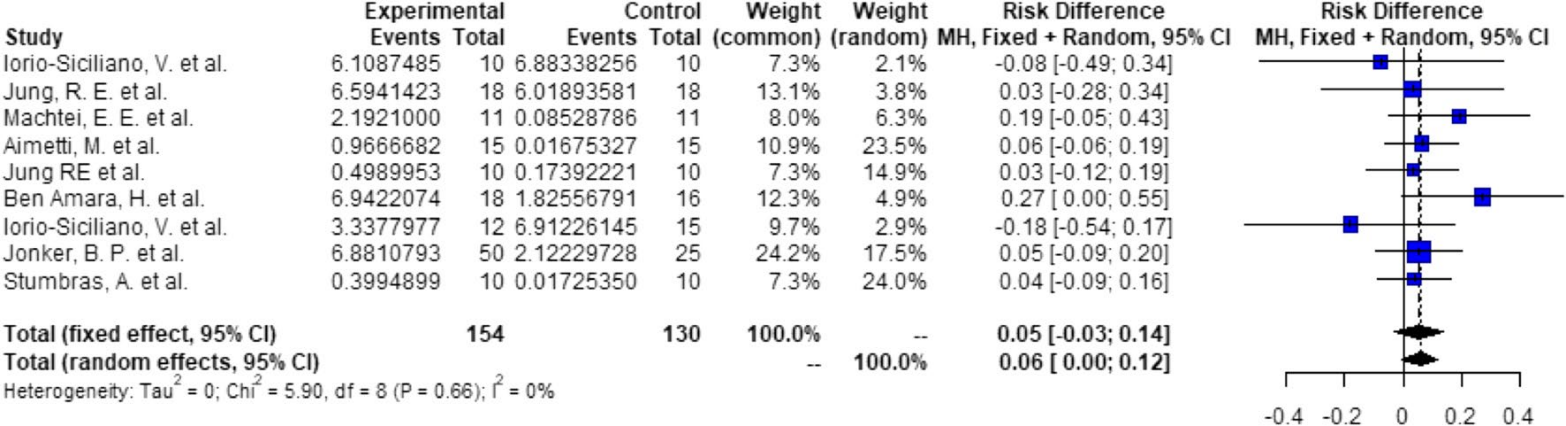
Meta-analysis results for the binary outcomes for implant placement in a bone envelope of 9.0 mm

1. Probabilities/events for 7 (1 + 5 + 1) mm:
  There is a significant higher probability of 21% (RD = 0.21, *C* = 0.06–0.35, *p* < 0.01), that after ARP using DBBM, 5 mm implants can be placed with 1 mm of circumferential bony housing allowing simultaneous GBR (Fig. 8).
2. Probabilities/events for 9 (2 + 5 + 2) mm:
  There is a non-significant higher probability of 6% (RD = 0.06, *C* = 0.00–0.12, *p* = 0.06), that after ARP using DBBM, 5 mm implants can be placed without any further bone grafting procedure with 2 mm of circumferential bony housing (Fig. 9).

Overall average probabilities based on the nine assessed RCTs are displayed in Tables 4, 5

**Table 4:** Overview of the probabilities to place a standard diameter implant in relation to a predefined bony housing

	1 + 3.25 + 1	2 + 3.25 + 2	1 + 4.0 + 1	2 + 4.0 + 2	1 + 5.0 + 1	2 + 5.0 + 2
Test (ARP)	0.82%	0.51%	0.73%	0.37%	0.55%	0.30%
Control (SH)	0.63%	0.32%	0.50%	0.26%	0.35%	0.19%

**Table 5:** Risk of bias assessment according to the Cochrane Collaboration’s tool (2011)

Author and year	Adequate sequence generation	Allocation concealment	Blinding	Incomplete outcome data addressed	Selective outcome reporting	Free of other sources of bias	Estimated potential risk of bias
Aimetti et al. 2018 [21]	Yes	Yes	Yes	Yes	Yes	Yes	Low risk
Ben Amara et al. 2021 [23]	Yes	Yes	Yes	Yes	Yes	Yes	Low risk
Iorio-Siciliano et al. 2017 [22]	Yes	Yes	No	Yes	Yes	Yes	Moderate risk
Iorio-Siciliano et al. 2020 [16]	Yes	Yes	No	Yes	Yes	Yes	Moderate risk
Jonker et al. 2021 [18]	Yes	Yes	Yes	Yes	Yes	Yes	Low risk
Jung et al. 2013 [17]	Yes	Yes	No	Yes	Yes	No	High risk
Jung et al. 2018 [15]	Yes	Yes	No	Yes	Yes	Yes	Moderate risk
Machtei et al. 2019 [19]	Yes	Yes	Yes	Yes	Yes	Yes	Low risk
Stumbras et al. 2021 [20]	Yes	Yes	Yes	Yes	Yes	Yes	Low risk

The studies meeting all of the criteria were classified as having a low risk of bias, while those that did not meet a criterion were classified as having moderate risk. When two or more criteria were not met, the studies were considered to have a high risk of bias

### Risk of bias in individual studies

The risk ranged from high to low risk throughout the included studies, as shown in Table 4. The most common missing characteristic was the blinding for outcome measures. One study reported of a significant higher number of smokers in one treatment group [17].

### Risk of bias across studies

**Fig. 10:**
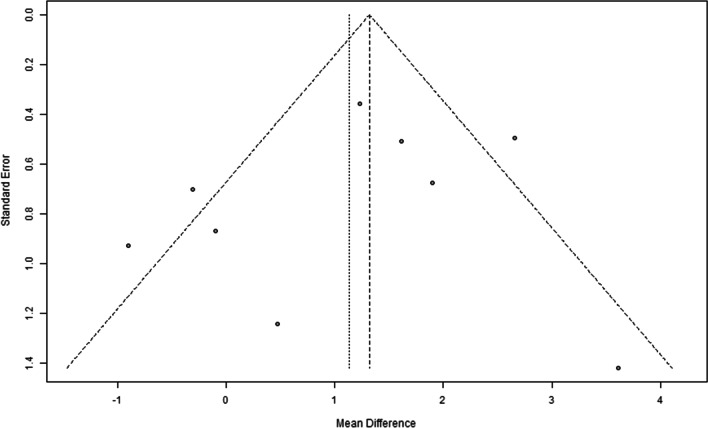
Funnel plot for the original outcome (bone crest in mm)

No significant publication bias was observed for the studies in terms of primary outcome following the funnel plots (Fig. 10).

### Number needed to treat

The NNT values calculated as 1/|RD| ranged between 4.5 and 5.5 in required bony envelopes from 5.25 to 7.0 and increased to 7.2 up to 17.3 in required bony envelopes of 8.0 and 9.0 mm, respectively. This means that we benefit in roughly every fifth patient from ARP, whereas this number even increases with larger implant diameters and bone widths.

## Discussion

Several systematic reviews and meta-analysis have shown that while no technique or biomaterial is able to completely eliminate post-extraction resorption, ARP will minimize especially horizontal soft and hard tissue shrinkage [5, 24–28]. Consequently, different ARP modalities based on clinical scenarios have been proposed to enable soft, hard or soft and hard tissue preservation, recently [29]. Maintaining the ridge contour by applying an ARP technique is only of secondary relevance, primary aim must be a long-term stable implant supported reconstruction. However, the clinical significance on the possibility to place an implant with “sufficient” bone or without additional bone grafting, long-term implant success or patient-oriented outcomes as treatment time, costs, etc., is still missing.

Applying DBBM during ARP reduces the dimensional changes after tooth extraction on average by 1.13 mm and, thereby, improves the possibility to place standard diameter implants with 2.0 mm bony housing up to 22% after 2 to 12 months. Noteworthy, NNTs depend on selected implant diameter and required bone housing. Roughly, every fifth patient will profit from ARP based on the above stated calculations; however, this means that 4 out 5 patients might not need bone augmentation after SH or might still require a second augmentation after ARP. Hence, though there is a statistically significant advantage of ARP over SH, the clinical benefit remains unclear. Furthermore, the cost–benefit of ARP needs to be discussed. The findings of the current study go along with the study by Mardas et al., indicating a decrease in the need for further ridge augmentation, when ARP was performed [4]. If minimizing alveolar ridge reduction, especially in horizontal dimension, is priority, ARP should be considered. Nevertheless, the impact on implant survival, marginal bone loss or susceptibility to peri-implant diseases remains unclear [5]. Future research needs to focus on patient centred outcomes as well as the long-term success of implants placed after ARP or staged bone reconstruction (like GBR or sinus grafting).

### Strengths and limitations

To the authors best knowledge, this is the first report to assess the statistical possibility to place different diameter implants with up to 2 mm surrounding bone after ARP. Since 2-mm bony housing have been proposed as the border between a thin or thick peri-implant phenotype recently [30], this might be regarded as a prerequisite for stable hard tissue over time. If this is enabled by applying ARP and no additional bone augmentation is needed, this might be seen as a truly clinical relevant endpoint. Nevertheless, when dealing with cases in the anterior zone, bone reconstruction might not only be warranted for functional but aesthetic reasons and, even in cases with > 2 mm surrounding bone, additional ridge corrections might be warranted to achieve a natural ridge curvature. Furthermore, while the calculated numbers might show the possibility to place an implant, it is not possible to assess whether or not an prosthetically driven implant position is feasible based on the included data especially since the major change in ridge dimension needs to be anticipated from the buccal [2]. Within the literature, often, it is not differentiated between bone augmentations needed to treat, e.g. a thin bone situations or dehiscence defects—functional aspects, < 2 mm bone—or to correct ridge contour deficiencies with implants surrounded with already > 2 mm of bone—aesthetic aspects [31]. Further confounding aspects not taken into consideration might be periodontal phenotype, socket configuration (intact versus deficient), reason of tooth extraction, flap reflection and attempting primary closure.

To reduce the heterogeneity of the included studies, only trials assessing the application of DBBM were selected. While reducing the number of confounding factors like different clinical outcomes related to the applied biomaterials, it also reduces the power of this systematic review and neglects the wide range of clinically applied bone substitutes. On the other side, uneven data exist for different biomaterials with the largest amount of studies for DBBM [32]. Two recent systematic reviews from the same research group focused on the effect of different grafting materials on ridge maintenance [24] and histomorphometric socket healing [33]. While xenografts including DBBM showed greater alveolar width and height preservation, major differences were observed for new bone formation between, e.g. bovine or porcine xenografts with the lowest percentage of new bone for particulate DBBM.

Bone width measurements have been undertaken at crest level or 1 mm below in eight out of nine studies, while one study assessed the width 3 mm below the crest. This represents a very comparable situation overall, especially in the main question assessed in the current review, as predominantly the region of the implant shoulder is a key-point for the necessity of additional bone augmentation procedures. Originally, we aimed to assess differences between anterior vs posterior and/or single-rooted vs multi-rooted teeth, however, due to the variances in the treatment protocols and presented data within the included studies, this was not feasible. This also accounted for assessing the effect of distinctive healing periods.

Although a comprehensive search strategy including five databases, it is possible that some grey literature may not have been included as only published studies in English language were selected. Furthermore, the authors of six studies selected for full-text screening were contacted via email to request further information relating to the dimensional changes following ARP, however, some authors failed to respond within the requested period of time (4 weeks). Therefore, it is probable that further information exists which could be used to complement the data set used in this review.

## Conclusions

Within the limitations of present systematic review, the following conclusions can be drawn:

1. ARP with DBBM significantly reduces the horizontal dimensional changes after tooth extraction.
2. ARP significantly improves the possibility to place standard diameter implants with at least 1 mm of bony housing.
3. ARP, thereby, potentially reduces the complexity of bone reconstruction and the need for further ridge augmentation during implant placement.

## Data Availability

All data generated or analysed during this study are included in this published article.
